# Itaconic acid inhibits nontuberculous mycobacterial growth in pH dependent manner while 4-octyl-itaconic acid enhances THP-1 clearance of nontuberculous mycobacteria *in vitro*

**DOI:** 10.1371/journal.pone.0303516

**Published:** 2024-05-10

**Authors:** Paul Breen, Madsen Zimbric, Lindsay J. Caverly

**Affiliations:** Department of Pediatrics, University of Michigan Medical School, Ann Arbor, MI, United States of America; Wayne State University School of Medicine, UNITED STATES

## Abstract

Increasingly prevalent, nontuberculous mycobacteria (NTM) infections affect approximately 20% of people with cystic fibrosis (CF). Previous studies of CF sputum identified lower levels of the host metabolite itaconate in those infected with NTM. Itaconate can inhibit the growth of *M*. *tuberculosis* (MTB) *in vitro* via the inhibition of the glyoxylate cycle enzyme (ICL), but its impact on NTM is unclear. To test itaconic acid’s (IA) effect on NTM growth, laboratory and CF clinical strains of *Mycobacterium abscessus* and *Mycobacterium avium* were cultured in 7H9 minimal media supplemented with 1–10 mM of IA and short-chain fatty acids (SCFA). *M*. *avium* and *M*. *abscessus* grew when supplemented with SCFAs, whereas the addition of IA (≥ 10 mM) completely inhibited NTM growth. NTM supplemented with acetate or propionate and 5 mM IA displayed slower growth than NTM cultured with SCFA and ≤ 1 mM of IA. However, IA’s inhibition of NTM was pH dependent; as similar and higher quantities (100 mM) of pH adjusted IA (pH 7) did not inhibit growth *in vitro*, while in an acidic minimal media (pH 6.1), 1 to 5 mM of non-pH adjusted IA inhibited growth. None of the examined isolates displayed the ability to utilize IA as a carbon source, and IA added to *M*. *abscessus* isocitrate lyase (ICL) decreased enzymatic activity. Lastly, the addition of cell-permeable 4-octyl itaconate (4-OI) to THP-1 cells enhanced NTM clearance, demonstrating a potential role for IA/itaconate in host defense against NTM infections.

## Introduction

*Mycobacterium* possess several distinct biological features: high lipid and mycolic acid abundance in their cell walls, extremely low cell permeability, high temperature resistance, acidic pH tolerance, and a slow cell doubling time [1–4]. While *Mycobacterium tuberculosis* (MTB) and *Mycobacterium leprae* are the most well-known species, nontuberculous mycobacterial (NTM) infections are growing in prevalence in both immunocompromised and immunocompetent individuals, particularly in those with underlying lung diseases [2,5]. Commonly referred to environmental mycobacteria, NTM are classified as any mycobacteria that do not cause tuberculosis or leprosy [6,7]. NTM are ubiquitous in the environment, particularly in water and soil, and are genetically diverse [8–10]. While NTM exposure is common, they rarely cause disease in healthy individuals [11,12]. Most human NTM pulmonary infections are caused by the “slow growing” mycobacteria such as the members of the *Mycobacterium avium* complex (MAC), which includes species such as *Mycobacterium avium*, *Mycobacterium intracellulare*, and *Mycobacterium chimaera* [2,6]. MAC infections primarily manifest as pulmonary infections, however, other conditions such as skin lesions, peripheral lymphadenopathy, and in extreme cases disseminated infections can occur [13–16]. Pulmonary infections with “rapid growing” NTM such as subspecies in the *Mycobacterium abscessus* complex (MABC) are also increasing in prevalence [2,17]. However, unlike MAC, recent evidence suggests that MABC may be transmissible from person- to- person [2,18]. Similar to other mycobacteria, MAC and MABC are extremely difficult to treat with antibiotics and other therapeutics due to their thick cell wall, biofilm formation, and high rates of antimicrobial resistance [1,2,19,20].

Both MAC and MABC have been shown to primarily infect immunocompromised individuals and those with underlying lung disease such as primary ciliary dyskinesia and cystic fibrosis (CF) [2,20,21]. NTM infections currently affect about 20% of people with CF (pwCF), and that number continues to rise [22–25]. Contributors to the risk of NTM infection in pwCF include impaired mucociliary clearance, structural lung disease (bronchiectasis), and the impact of mutations in the cystic fibrosis transmembrane conductance regulator (CFTR) protein on NTM host response (e.g., impaired neutrophil and macrophage function, which are essential for responding to bacterial infections and can serve as a replication site for NTM) [26–31]. Additionally, individuals with a lower body mass index (with or without CF) seem to be at higher risk for NTM infection [21,24]. Related to lower body fat levels are lower levels of the protein leptin, which is involved in promoting Th1-mediated immunity (a significant component of the immune response to NTM infection), specifically with regulating CD4 numbers, interferon-γ expression, and T-cell function [21,32]. Studies have identified that leptin levels are significantly lower in the serum from pwCF compared to people without CF, that leptin levels are further decreased in pwCF that live a more sedentary lifestyle, and that decreased leptin levels result in impaired clearance of *M*. *abscessus* and *M*. *tuberculosis* [33,34].

The immune response to NTM in the lungs is initiated by alveolar macrophages, which are generally the first responders of the immune system, recognizing specific molecular patterns associated with NTM, primarily via toll-like receptors (TLRs) [21,35,36]. The interaction between the TLRs and NTM activates macrophages and induces proinflammatory cytokine production [21,37]. Macrophages play a critical role in regulating the host immune response to NTM infections through direct killing of NTM and production of IL-12, which activates natural killer or T cells to secrete IFN- γ [6,21]. In conjunction with IL-12, IFN- γ enables the initiation of the adaptive immune response to NTM pulmonary infections [21,38]. The activation of macrophages by lipopolysaccharide (LPS) and/or interferons also increases expression of the enzyme aconitate decarboxylase 1, also called immune-responsive gene 1 protein (IRG1), causing cis-aconitate to be diverted from the TCA cycle and repurposed for itaconate production [39,40]. The secretion of itaconate from macrophages is a key marker of macrophage antibacterial response [40–42]. While the role of itaconate in NTM pulmonary infection is unclear, several recent studies have examined the direct effects of itaconate on other pathogenic bacteria, as the compound has been shown to have antimicrobial and anti-inflammatory properties [40,43,44]. Specifically, itaconate inhibits multiple bacterial enzymes including isocitrate lyase (ICL), succinate dehydrogenase (SDH) and propionyl-CoA carboxylase [40–42,45,46]. A recent study by Demars *et al*. demonstrated that itaconate can inhibit the growth of *Brucella* in a dose dependent manner, likely via inhibition of the glyoxylate cycle enzyme ICL [47]. While itaconate seems to have more of an antimicrobial role with certain bacterial pathogens, in contrast, studies in CF have found that itaconate promotes the formation of biofilms in *Staphylococcus aureus*, and that *Pseudomonas aeruginosa* can utilize itaconate for growth [41,48,49].

Itaconate’s anti-inflammatory effect could be of particular relevance for NTM infection, as excessive immune responses can hinder NTM clearance, allowing for increased bacterial proliferation [50]. Numerous studies have demonstrated that MTB elicits an immune response leading to supraphysiological concentrations of itaconate in macrophages [45,51,52]. However, the direct effects of itaconate on mycobacteria are mixed as MTB’s reaction to itaconate has shown conflicting results. Some studies have found that itaconate can inhibit MTB proliferation by inhibiting the glyoxylate cycle enzyme, ICL [45,53,54]. Importantly, itaconate inhibits the growth of MTB when the bacteria are in minimal medium supplemented with short chain fatty acids (SCFAs), compounds which are one of the preferred energy sources for mycobacteria inside of a host cell and require the ICL enzymes for utilization [55–58]. However, other studies have shown that MTB can dissimilate large quantities of itaconate into pyruvate and acetyl-CoA via the enzyme Rv2498c [40,59]. While there is published data available on how itaconate affects MTB, the same is not true of NTM species, where limited data are available. A previous study from our lab demonstrated that sputum itaconate levels are decreased in pwCF prior to and during NTM infection, compared to those without NTM infection [60]. Additionally, the amount of itaconate needed to inhibit bacterial growth may be dependent on environmental factors, specifically pH, as recent studies suggest there may be a synergy between the effectiveness of itaconate and pH [47,61]. Given the demonstrated direct interactions between itaconate and MTB, this suggests that a mechanistic relationship may exist between the compound and clinically isolated strains of pulmonary NTM that needs to be explored.

In this study, we examined the effects of itaconic acid (IA) on MAC and MABC isolate growth and macrophage clearance. MAC and MABC reference strains and CF clinical isolates were able to grow when supplemented with SCFAs but the addition of IA at ≥ 10 mM completely inhibited the growth of all NTM. NTM supplemented with acetate or propionate and 5 mM IA displayed a slower rate of growth than NTM cultured with either of these two SCFAs and 1 mM of IA. However, IA’s inhibition of NTM was pH dependent; as similar and higher quantities (100 mM) of pH adjusted IA (pH 7) did not inhibit NTM growth in vitro. Alternatively, in a more acidic minimal media (pH 6.1) akin to the pH of the CF airway [62–64], lower quantities of non-pH adjusted IA (1–5 mM) inhibited NTM growth. None of the examined NTM isolates displayed the ability to utilize IA as a carbon source and IA inhibited the enzyme ICL. Lastly, the addition of 4-OI, a membrane permeable form of itaconate, to differentiated THP-1 cells enhanced the clearance of phagocytosed bacteria *in vitro*. Our results indicate that IA/itaconate can inhibit the growth and clearance of MAC and MABC, suggesting that this compound plays an important role in the host response to NTM infections.

## Materials and methods

### Bacterial strains and culture conditions

The NTM strains used in this study are listed in Table 1. All clinical samples are from CF patients while lettered samples are mixed morphology cultures from the same date and same patient. Bacteria were cultured at 37° C in 7H9 broth with OADC (Remel, R450605). For minimal media experiments, a modified 7H9 broth was used based on the methods described by Muñoz-Elías and McKinney [58], specifically, NTM were grown in 7H9 + 0.5% albumin, 0.085% NaCl, 0.05% Tween-80, and carbon substrate (10 mM short chain fatty acid) for the indicated number of days. For the pH adjusted minimal media, the media with all the above listed components was pH adjusted and then filter sterilized prior to SCFA addition and use. IA (Sigma-Aldrich, I29204) was resuspended in sterile ddH2O, pH adjusted (for the pH = 7 IA), filter sterilized, and added to the 7H9 media to the desired concentration. For pH-neutral IA experiments, IA received NaOH to bring the pH to a neutral range before being brought to the desired final volume and filter sterilized for addition to the 7H9 media. 4-Octyl itaconate (MedChem Express, HY-112675) was resuspended in DMSO and added to THP-1 cells at the desired concentrations. The short chain fatty acids (SCFA) acetate, propionate, and butyrate (Sigma-Aldrich, S2889, P5436, & 303410, respectively) were resuspended in sterile ddH20 and filter sterilized prior to use.

**Table 1:** List of NTM isolates used in this study.

NTM Isolate	Species	Morphology	Specimen source	NCBI Biosample^1^
**ATCC 19977**	***M*. *abscessus***			
**ATCC 25291**	***M*. *avium***			
**FLAC0253a**	***M*. *abscessus complex***	**Smooth**	**Bronchoalveolar lavage**	**SAMN35656211**
**FLAC0253b**	***M*. *abscessus complex***	**Rough**	**Bronchoalveolar lavage**	**SAMN35656212**
**FLAC0711a**	***M*. *abscessus complex***	**Smooth**	**Sputum**	**SAMN35656215**
**FLAC0711b**	***M*. *abscessus complex***	**Rough**	**Sputum**	**SAMN35656216**
**FLAC1243**	***M*. *abscessus***	**Smooth**	**Sputum**	**SAMN35656227**
**FLAC0493**	***M*. *abscessus complex***	**Rough**	**Sputum**	**n/a**
**FLAC1238**	***M*. *abscessus***	**Rough**	**Sputum**	**SAMN35656225**
**FLAC0622**	***M*. *avium complex***	**Smooth**	**Sputum**	**SAMN35656214**
**FLAC0813**	***M*. *avium complex***	**Smooth**	**Sputum**	**SAMN35656217**
**FLAC1071**	***M*. *chimaera-intracellulare***	**Smooth**	**Sputum**	**SAMN35656220**
**FLAC1151**	***M*. *chimaera-intracellulare***	**Smooth**	**Sputum**	**n/a**

^1^ Samples associated with BioProject PRJNA315990.

### Cell culture conditions

THP-1 cells were maintained in RPMI (Gibco, 22400–089) supplemented with 10% FBS (Corning, 35-010-CV) and 1% Penn/strep (Fisher Scientific, 15140122) and incubated at 37°C with 5% CO2 in a water-jacketed incubator (ThermoForma, Model 3130). THP-1 cells were differentiated using 5 ng/mL PMA (Sigma-Aldrich, P1585) and seeded into a 24 well plate (Fisher Scientific, FB012929) at a concentration of 2*10^5^ cells/mL for 24 hours. Following differentiation, the cell media was aspirated off and fresh media was added to the cells without antibiotics for the *M*. *abscessus* experiments or with chloramphenicol (20 ug/mL) for experiments with *M*. *avium* due to the long incubation period. Following a 72-hour recovery, NTM and 4-OI was added to the cells at the indicated MOIs and concentrations then incubated as described above. No pH adjustments were made to the 4-OI or the cell culture media. To determine the number of surviving NTM colonies, at the desired time points the 24-well plates were removed from incubation, the media was aspirated off and a lysing buffer of 0.05% SDS in sterile PBS was added to the monolayer to lyse THP-1 cells. Cell lysates were then serially diluted and plated for NTM enumeration on Middlebrook 7H10 agar plates.

### Minimal media experiments

NTM grown to stationary phase in 7H9 broth supplemented with OADC were washed with 1x PBS, and resuspended in 7H9 minimal media with the indicated SCFA to a final concentration of 1 x 10^6 CFU/mL. Cultures were incubated in 14 mL snap cap tubes (Corning, 352006) at 37° C for the indicated time periods with OD_600_ readings taken daily using sterile technique to track bacterial growth using a SpectraMax M2^e^ Spectrophotometer with SoftMax Pro V7.1 software.

### ICL assay

Isocitrate lyase (ICL) activity was measured based on the protocol described by Nguyen *et al*. [65] while the preparation of cell extracts was based on that of Bentrup *et al*. [54]. Briefly, *M*. *abscessus* samples were grown to stationary phase and then diluted to an OD_600_ of ~0.9–1. Following the dilution, 3 mL of the culture was centrifuged in a bench top centrifuge (Eppendorf 5415 D) at 9300g for 10 minutes, then washed in PBST (0.05% Tween 80) and resuspended in 1 mL of buffer containing 50 mM MOPS buffer (BP2900-500 Fisher Scientific), 5 mM MgCl2 (Sigma-Aldrich, M8266), 5 mM l-cysteine (Fisher Scientific, A10435-18), 1 mM EDTA (Invitrogen, 15575–038), and 50 uL of protease inhibitor cocktail (Millipore Sigma, P8465) prepared following manufactures instructions. The cells were disrupted with a mini Bead-Beater (Biospec Products) for two minutes at maximum speed and placed immediately on ice. The supernatant was harvested after centrifugation at 4°C for 10 min at max speed (9300 g) and stored at −80°C.

To quantify ICL activity, a buffer consisting of 0.50 mL of 50 mM imidazole buffer (Fisher Scientific, J67055,), 0.1 mL of 50 mM magnesium chloride solution (MgCl2), 0.1 mL of 10 mM ethylenediaminetetraacetic acid solution (EDTA), 0.1 mL of 40 mM phenylhydrazine hydrochloride solution (Millipore Sigma, 114715) and 0.1 mL of 10 mM DL-isocitric acid solution (isocitrate; Fisher Scientific, 205010010) was prepared and added into cuvettes (VWR, 58017–847), which were then equilibrated to 30°C. Following equilibration, 0.1 mL of thawed cell lysate and itaconate (where appropriate) was combined with the reagent mix and immediately measured in a spectrophotometer at 340 nm. Samples were placed back in the 30°C incubation and re-measured after 5 min for up to 20 minutes. Additional buffer was used as a sample blank. The standard curve was generated using the provided standard from an ICL assay kit (Mybiosource, MBS8243212) following the manufactures instructions.

### Statistical analysis

Two-way analyses of variance (ANOVA) with Tukey’s multiple-comparison test were conducted to test for significance. For the ICL assays, a paired-sample t-test was performed. Analyses were performed using GraphPad Prism 7.0, and Excel software.

## Results

### Itaconic acid inhibits NTM growth

**Fig 1:**
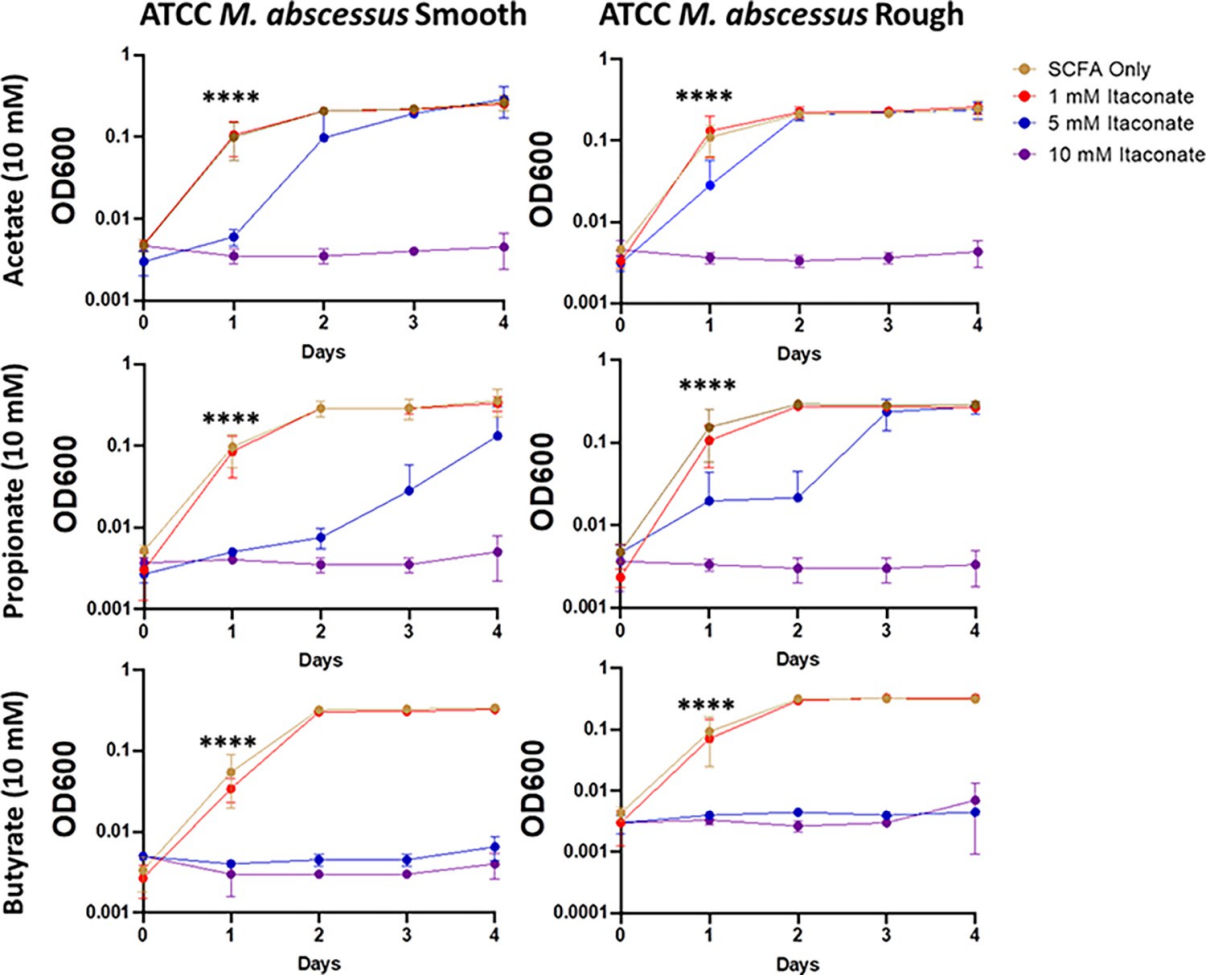
Itaconic acid inhibits the growth of ATCC *M*. *abscessus*. Smooth and rough ATCC *M*. *abscessus* was grown in 7H9 minimal media supplemented with 10 mM SCFAs: Acetate; propionate; or butyrate. IA was added at the indicated concentrations and an OD600 reading was taken each day to track bacterial growth. Statistical tests were performed using Two-Way ANOVA; Tukey’s multiple comparison test, on log transformed data. Error bars indicate standard deviation and significance indicates where the SCFA control was first found to be significantly different from 10 mM Itaconic acid samples. * p ≤ 0.05. ** p ≤ 0.005, ** p ≤ 0.0005, **** p ≤ 0.0001. n = 3.

**Fig 2:**
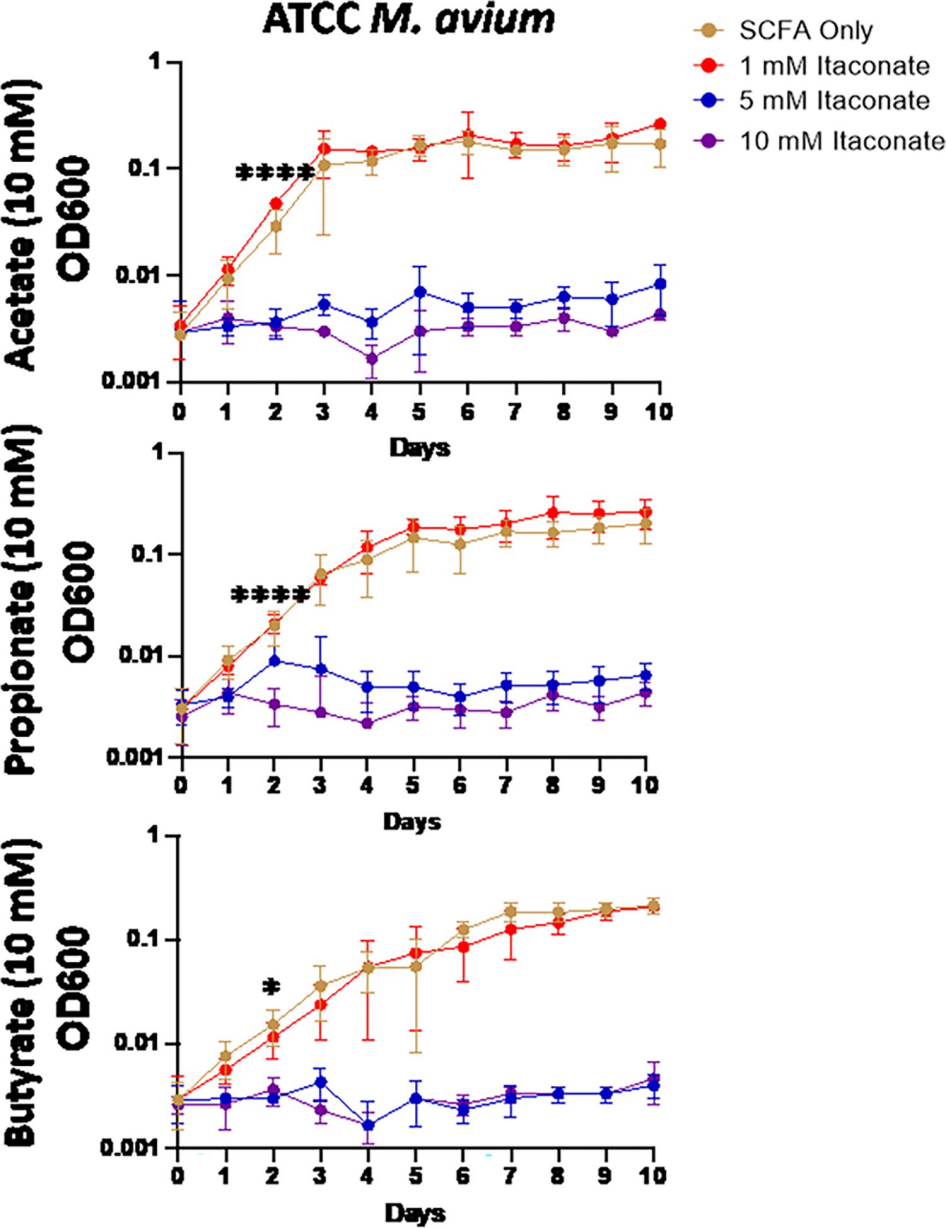
Itaconic acid inhibits the growth of ATCC *M*. *avium*. ATCC *M*. *avium* was grown in 7H9 minimal media supplemented with 10 mM SCFAs: Acetate; propionate; or butyrate. IA was added at the indicated concentrations and an OD600 reading was taken each day to track bacterial growth. Statistical tests were performed using Two-Way ANOVA; Tukey’s multiple comparison test, on log transformed data. Error bars indicate standard deviation and significance indicates where the SCFA control was first found to be significantly different from 10 mM Itaconic acid samples. * p ≤ 0.05. ** p ≤ 0.005, ** p ≤ 0.0005, **** p ≤ 0.0001. n = 2–5.

**Fig 3:**
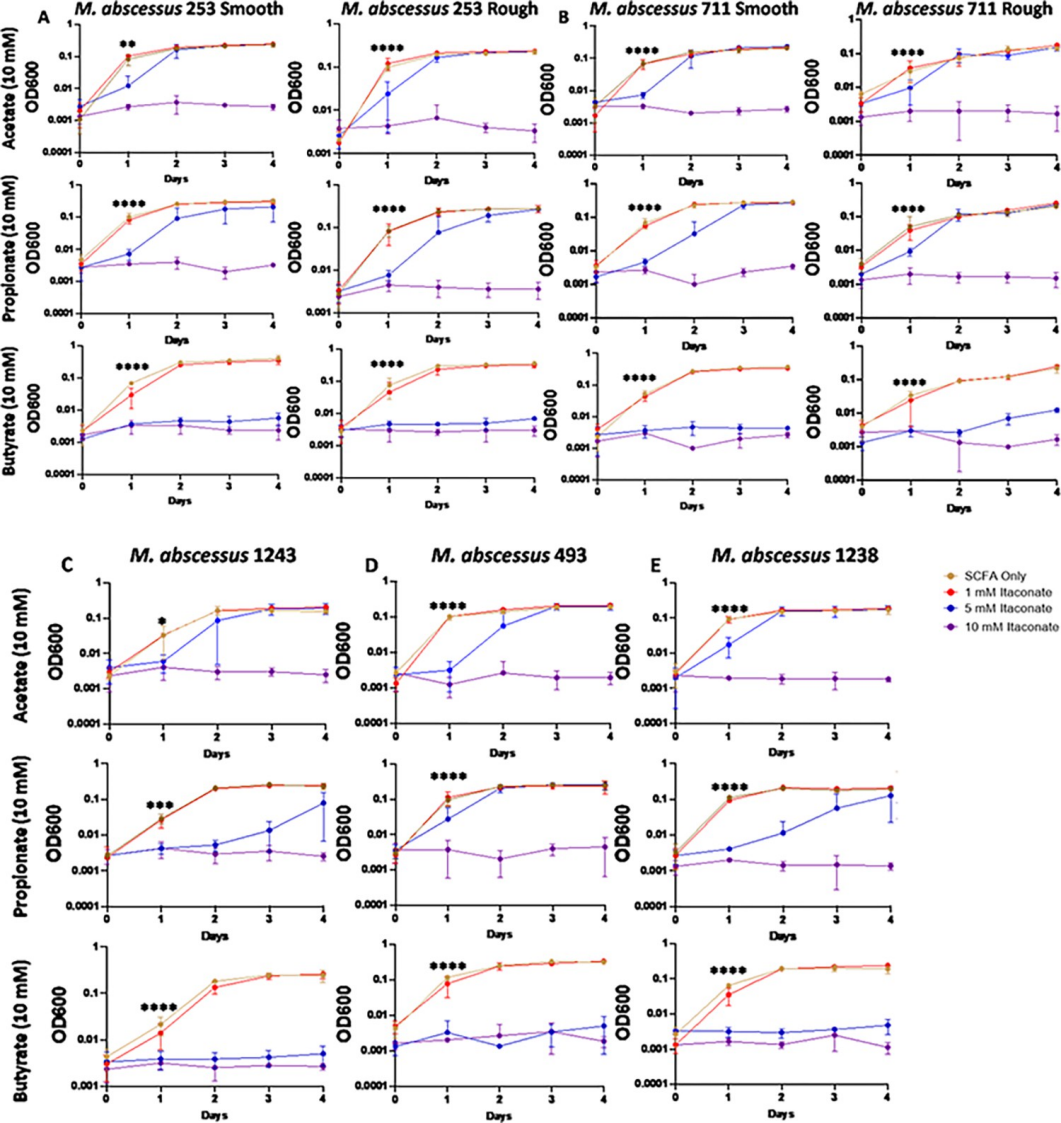
Itaconic acid inhibits the growth of clinically isolated *M*. *abscessus*. Smooth and rough clinical isolates of *M*. *abscessus* (A) 253, (B) 711, (C) 1243, (D) 493, (E) 1238, were grown in 7H9 minimal media supplemented with 10 mM of the indicated SCFAs. IA was added at the indicated concentrations and an OD600 reading was taken each day to track bacterial growth. Statistical tests were performed using Two-Way ANOVA; Tukey’s multiple comparison test, on log transformed data. Error bars indicate standard deviation and significance indicates where the SCFA control was first found to be significantly different from 10 mM Itaconic acid samples. * p ≤ 0.05. ** p ≤ 0.005, ** p ≤ 0.0005, **** p ≤ 0.0001. n = 2–5.

**Fig 4:**
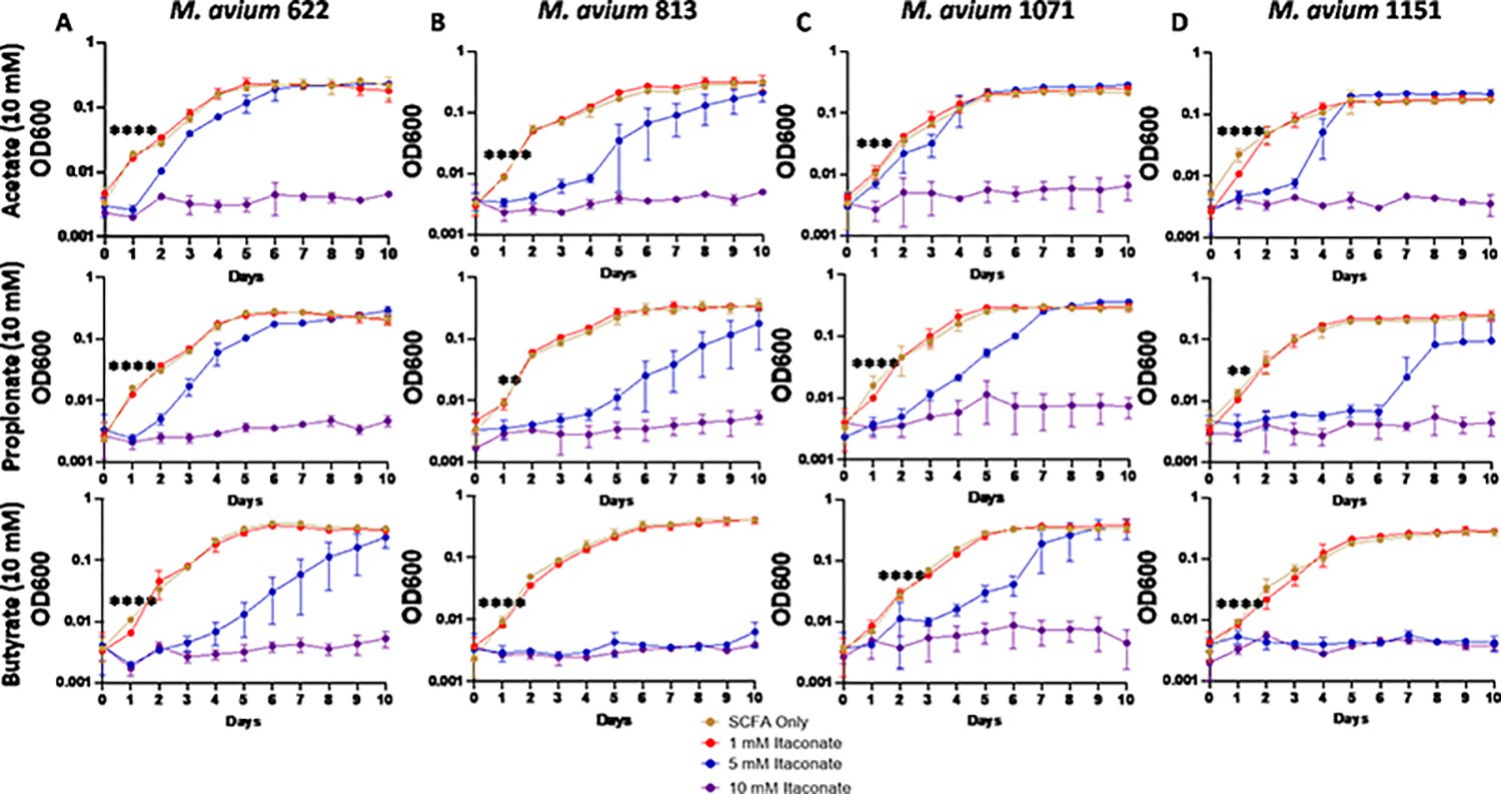
Itaconic acid inhibits the growth of clinically isolated MAC. Clinical isolates of MAC (A) 622, (B) 813, (C) 1071, (D) 1151, were grown in 7H9 minimal media supplemented with 10 mM of the indicated SCFAs. IA was added at the indicated concentrations and an OD600 reading was taken each day to track bacterial growth. Statistical tests were performed using Two-Way ANOVA; Tukey’s multiple comparison test, on log transformed data. Error bars indicate standard deviation and significance indicates where the SCFA control was first found to be significantly different from 10 mM Itaconic acid samples. * p ≤ 0.05. ** p ≤ 0.005, ** p ≤ 0.0005, **** p ≤ 0.0001. n = 2–3.

**Fig 5:**
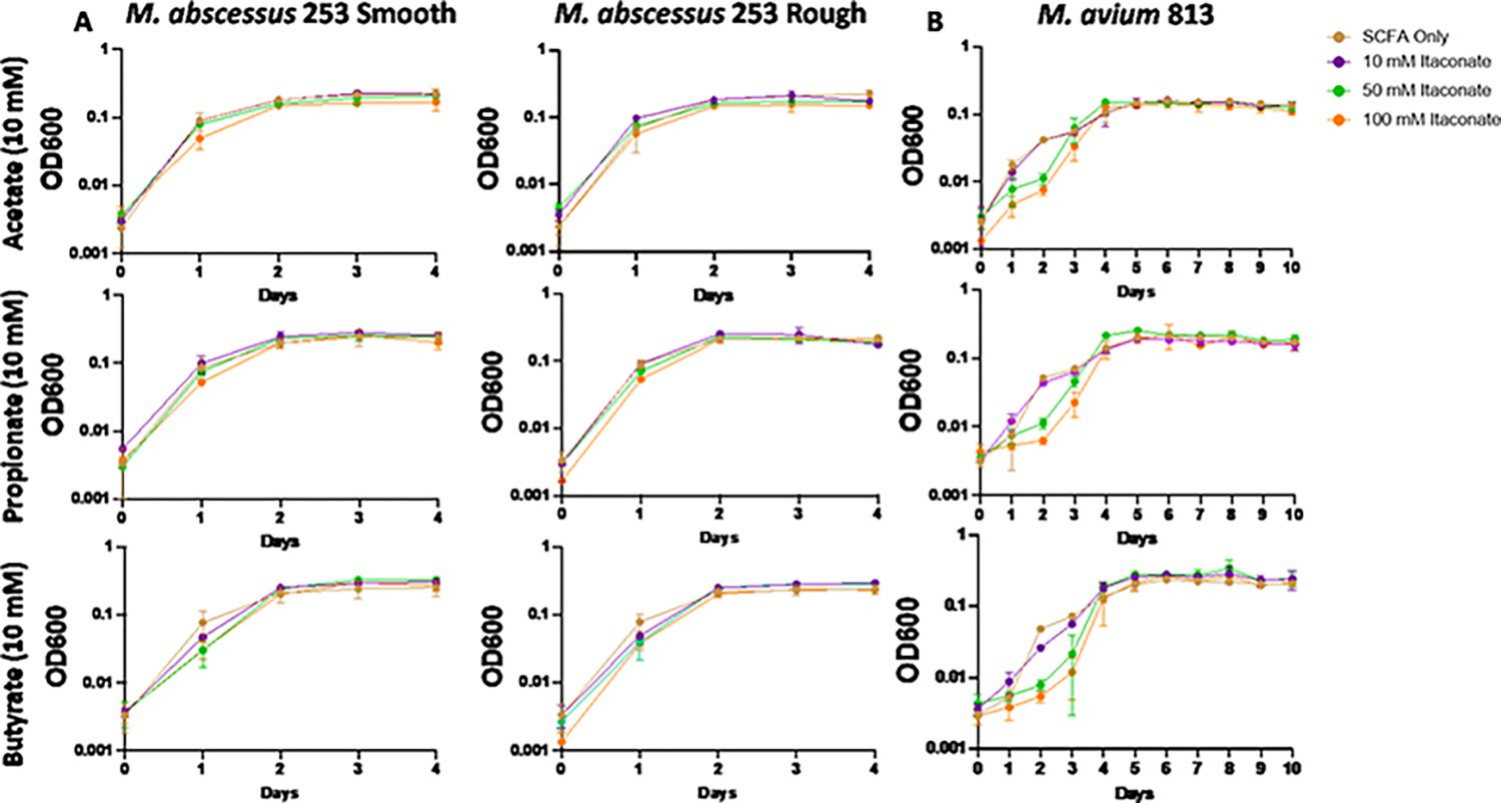
pH neutral itaconic acid does not inhibit the growth of *M*. *abscessus* and *M*. *avium*. Clinically isolated (A) *M*. *abscessus* 253 and (B) *M*. *avium* 813 was grown in 7H9 minimal media supplemented with 10 mM SCFAs: Acetate, propionate, and butyrate. IA (pH≈7) was added at the indicated concentrations and an OD_600_ reading was taken each day to track bacterial growth. Statistical tests were performed using Two-Way ANOVA; Tukey’s multiple comparison test, on log transformed data. Error bars indicate standard deviation and significance indicates where the SCFA control was first found to be significantly different from 10 mM Itaconic acid samples. * p ≤ 0.05. ** p ≤ 0.005, ** p ≤ 0.0005, **** p ≤ 0.0001. n = 3–5.

**Fig 6:**
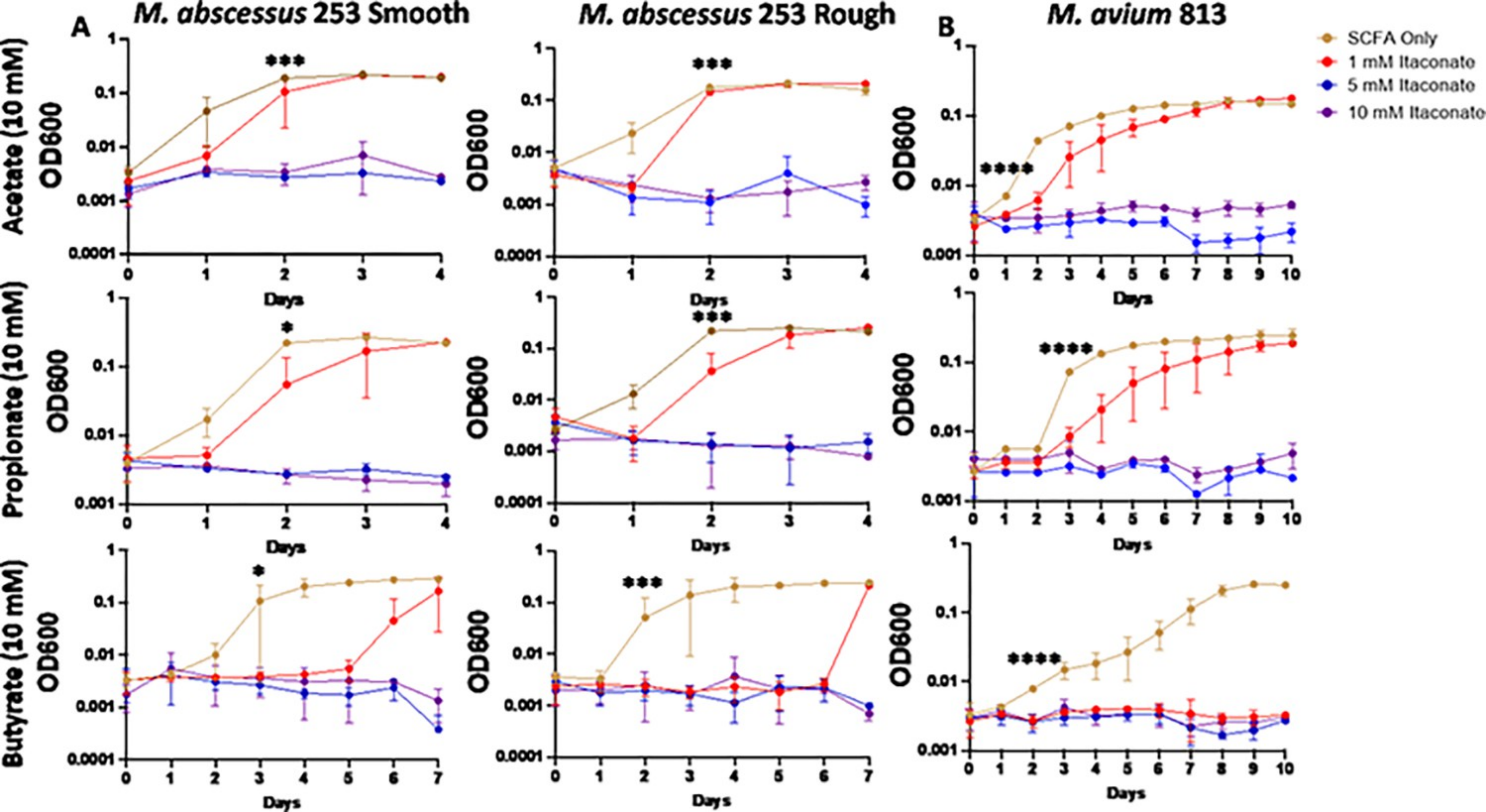
Increased acidity enhances the inhibitory effects of itaconic acid on *M*. *abscessus* and *M*. *avium*. Clinical isolates of (A) smooth and rough *M*. *abscessus* 253 and (B) *M*. *avium* 813 was grown in 7H9 minimal media adjusted to pH = 6.1 supplemented with 10 mM SCFAs: Acetate, propionate, and butyrate. IA was added at the indicated concentrations and an OD600 reading was taken each day to track bacterial growth. Statistical tests were performed using Two-Way ANOVA; Tukey’s multiple comparison test, on log transformed data. Error bars indicate standard deviation and significance indicates where the SCFA control was first found to be significantly different from 10 mM Itaconic acid samples. * p ≤ 0.05. ** p ≤ 0.005, ** p ≤ 0.0005, **** p ≤ 0.0001. n = 3–6.

Studies examining the effects of itaconate on MTB demonstrate that itaconate can inhibit the growth of some strains of MTB while others can metabolize the compound [59]; however, the effects of itaconate on NTM growth have been mostly unevaluated. To examine this, we cultured a total of 5 MAC and 6 MABC isolates (Table 1) in 7H9 minimal media (MM) supplemented with one of three SCFAs NTM would likely utilize as a carbon source during an infection, acetate (C2), propionate (C3), or butyrate (C4). Different SCFAs were used to determine if the length of the SCFA affected IA inhibition. All MAC and MABC NTM isolates cultured in minimal media with SCFA supplementation were able to proliferate (Figs 1–6), with MABC isolates generally reaching stationary phase within 3 days (Figs 1 and 3) and MAC isolates generally reaching stationary phase by day 6 (Figs 2 and 4). In all examined isolates, the addition of 10 mM of IA prevented any bacterial growth (Figs 1–4). Significant differences in the rate of cell proliferation between SCFA control and 10 mM IA could often be observed after 1 day in all tested MABC isolates (Figs 1 and 3), while for MAC, significant differences in growth were observed by day 1 in most tested isolates (isolates 622, 813, 1071 grown with acetate and propionate, and 1171), while for some isolates significant differences were not observed till day 2 (ATCC, isolate 1071 grown with butyrate) (Figs 2 and 4). The *M*. *avium* ATCC strain was unable to grow with the addition of 5 mM IA when supplemented with either propionate or butyrate (Fig 2). MABC isolates cultured in MM containing butyrate and 5 mM of IA were unable to proliferate (Figs 1 and 3); this was also true for three of the five MAC strains with the exceptions of strains 622 and 1071, which were able to grow in those conditions (Figs 2, 4A and 4D). We also observed that in MM containing either acetate or propionate and 5 mM of IA the proliferation of NTM was delayed compared to NTM cultured with 1 mM of IA, specifically, the amount of growth on day 1 was typically a log lower than the NTM grown in MM with SCFA alone or 1 mM IA, however by the end of the experimental measurements, NTM cultured with 5 mM IA was able to reach an OD comparable to the NTM grown in MM with SCFA alone or SCFA and 1 mM IA.

### Itaconic acid is highly acidic and its inhibition of NTM is pH dependent

While our results demonstrate that IA can inhibit the growth of NTM, recent studies examining the inhibitory properties of itaconate have also begun to consider the role of its highly acidic pH in that inhibition [61]. Indeed, the addition of IA to the 7H9 minimal media (initial pH = 6.6) altered the final pH values of the media (1 mM IA final pH = 6.5; 5 mM IA final pH = 5.7; 10 mM IA final pH = 4.8). *M*. *abscessus* 253 and *M*. *avium* 813 cultured in pH = 4.8 7H9 minimal media were able to proliferate, though at a lower rate, indicating that pH alone is not responsible for NTM inhibition (S1 Fig). To test the importance of pH in IA’s inhibitory role, two of the previously examined NTM strains (*M*. *abscessus* 253 and *M*. *avium* 813) were cultured in MM with SCFA and pH 7-adjusted IA. Under these conditions, 10 mM of IA was unable to inhibit the growth of the two NTM isolates tested (Fig 5). Furthermore, higher doses of pH neutral IA (50 and 100 mM) were also unable to inhibit the growth of the two NTM isolates tested (Fig 5). These results indicate that an acidic pH is an essential component to IA’s inhibitory properties.

### A lower media pH enhances itaconic acid’s inhibitory properties

After observing that pH neutral IA loses its inhibitory properties on NTM, we next sought to examine if a more acidic minimal media (pH = 6.1; a pH akin to the potentially more acidic pH found in the CF airway) would enhance the inhibitory properties of IA. Both tested NTM isolates (*M*. *abscessus* 253 and *M*. *avium* 813) were able to proliferate in pH 6.1 MM supplemented with SCFAs (Fig 6). However, under more acidic conditions, the inhibitory properties of IA were further enhanced; with 5 mM IA sufficient to limit the growth of all NTM isolates tested (Fig 6). Additionally, *M*. *abscessus* 253 cultured with butyrate and 1 mM IA took 6–7 days to begin proliferating (Fig 6A), while the *M*. *avium* 813 cultured in pH 6.1 MM with butyrate was unable to proliferate with the addition of 1 mM IA (Fig 6B).

### Itaconic acid inhibits the enzyme ICL in *M*. *abscessus*

**Fig 7:**
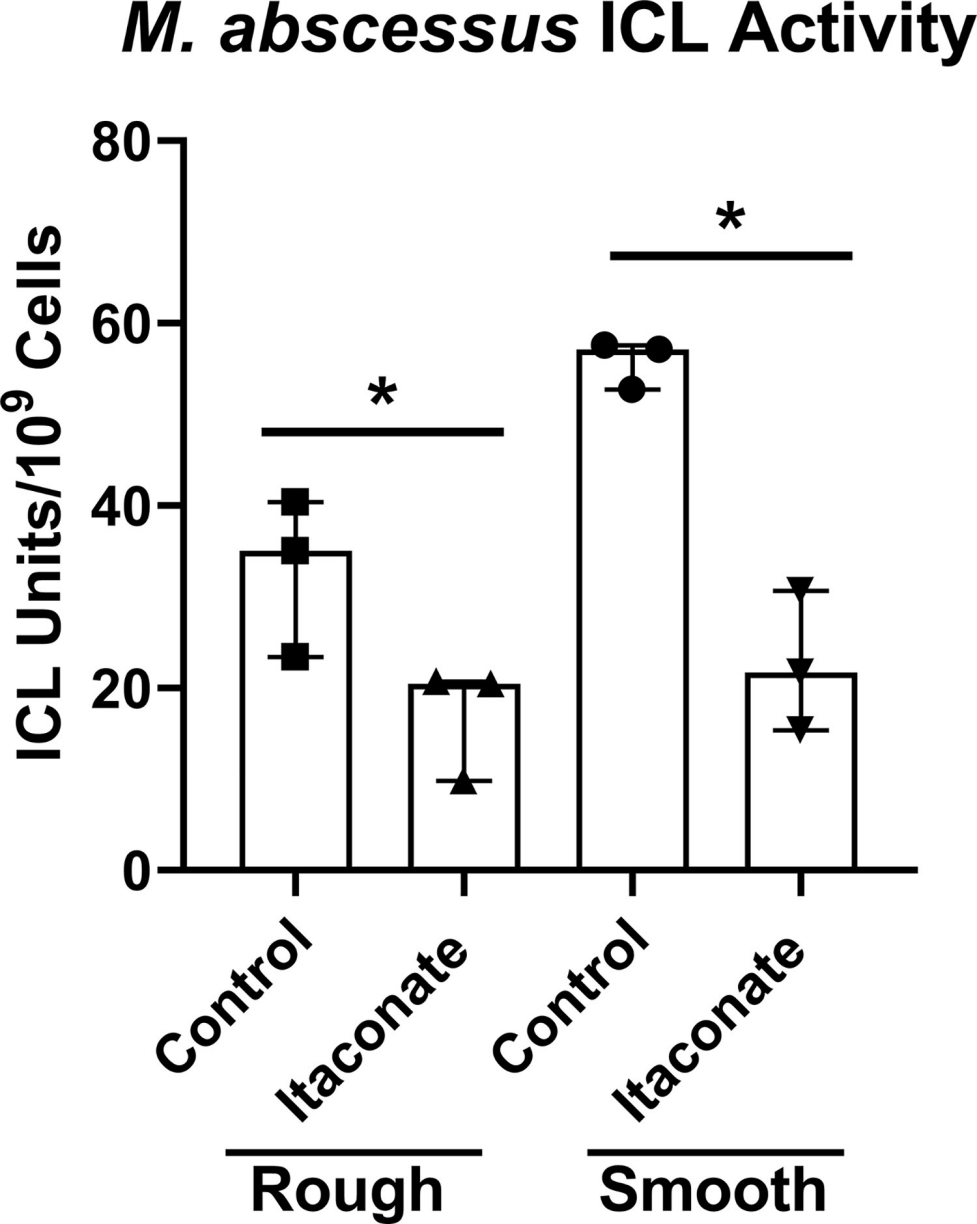
Itaconic acid reduces the enzymatic activity of ICL in *M*. *abscessus*. ATCC isolates of smooth and rough *M*. *abscessus* 253 grown in 7H9 media to stationary phase, collected, and lysed to acquire the bacterial ICL enzymes. IA (10 mM) was added to the cell lysate solutions and an OD_340_ reading was taken 20 minutes post addition to track enzymatic activity compared to the untreated controls. Statistical tests were performed using a paired t-test on log transformed data. Error bars indicate median with interquartile range and significance indicates where the SCFA control was first found to be significantly different from 10 mM Itaconic acid samples.* p ≤ 0.05. n = 3.

ICL activity is essential for the growth of MTB and *M*. *avium in vivo* [54,58,66]. Previous studies have shown that itaconate can inhibit ICL in both MTB and *M*. *avium* [54,67]. While the same is likely true for *M*. *abscessus*, it is unclear whether itaconate is causing ICL inhibition in this NTM species. As such, we sought to demonstrate that itaconate/IA is able to inhibit the activity of ICL within *M*. *abscessus*. The enzymatic activity of the ICL enzymes isolated from lysed from rough and smooth *M*. *abscessus* 253 were significantly reduced by the addition of 10 mM IA *in vitro* (Fig 7).

### 4-Octyl itaconate enhances the killing of NTM by differentiated THP-1 cells

**Fig 8:**
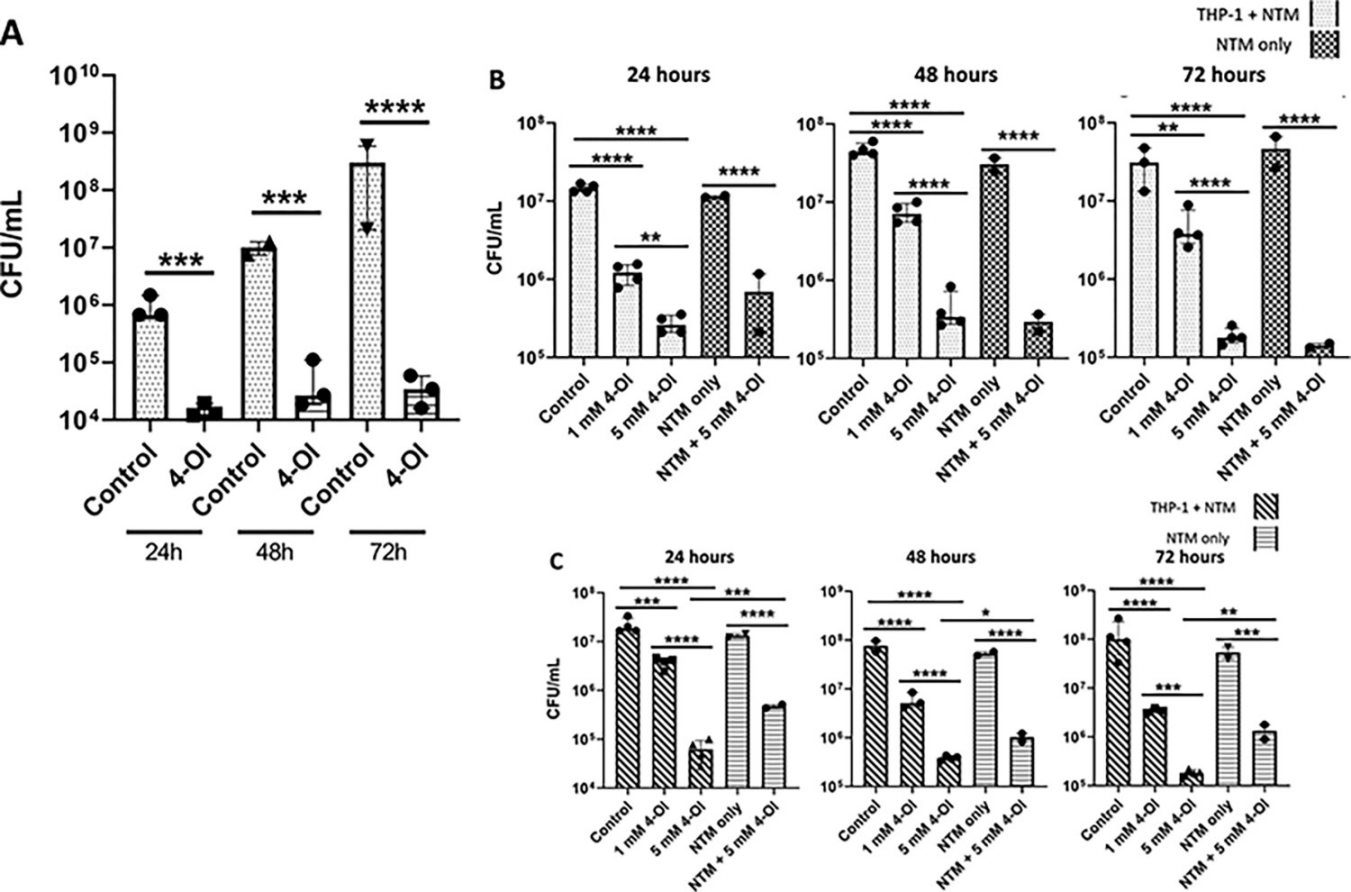
4-OI enhances the clearance of MABC by THP-1 cells. Clinically isolated *M*. *abscessus* 253 was added to differentiated THP-1 cells at an MOI of (A) 5:1 Smooth; (B) 5:1 Rough; and (C) 15:1 Smooth. 4-OI was added at the indicated concentrations (5 mM 4-OI for panel A) and cells were incubated at 37°C at 5% CO_2_ for the indicated lengths of time. Control refers to THP-1 cells exposed to NTM without the addition of any 4-OI. Statistical tests were performed using Two-Way ANOVA; Sidak’s multiple comparisons test, on log transformed data. Error bars indicate median with interquartile range and asterisks indicates a significant difference between the compared samples. **** p ≤ 0.0001. n = 2–4.

Based on our previous results demonstrating that IA can inhibit the growth of NTM, we next wanted to determine if adding itaconate to THP-1 cells would enhance their ability to eliminate NTM. Because itaconate is not cell-permeable, 4-octyl itaconate (4-OI), its cell-permeable derivative, was used in experiments as a substitute [68]. At the multiplicity of infections (MOIs) examined (5:1; 15:1), the THP-1 cells exposed to *M*. *abscessus* 253 and treated with 5 mM of 4-OI displayed a significantly higher rate of NTM clearance compared to the untreated controls. For the THP-1 cells exposed to MABC, a significant difference was recorded after 24 hours, with roughly a two-log difference observed in the number of surviving MABC (Fig 8A and 8B). The gap in surviving NTM between the 4-OI treated and untreated THP-1 cells further increased over the 48 and 72 hour time points, at maximum a nearly four-log difference was observed in the surviving smooth MABC between the 5 mM 4-OI treated THP-1 cells and the untreated THP-1 cells (Fig 8A). For the rough MABC, NTM clearance was 4-OI dose-dependent. Significant differences in number of surviving NTM were observed between 1 mM 4-OI treated THP-1 cells and untreated THP-1 cells, and between 5 mM 4-OI treated THP-1 cells and 1 mM 4-OI treated THP-1 cells at all examined time points (Fig 8B). No significant differences were observed when comparing the NTM alone to the THP-1 cells that were exposed to NTM or when comparing the NTM treated with 5 mM 4-OI against the THP-1 cells treated with 5 mM 4-OI and exposed to NTM (Fig 8B).

Due to the higher level of NTM clearance observed in the smooth MABC phenotype, we also examined THP-1 cells’ ability to clear smooth MABC at a 15:1 MOI. As observed with the THP-1 cells exposed to MABC at a 5:1 ratio, THP-1 cells that received either 1 or 5 mM of 4-OI cleared significantly more smooth MABC at all time points (Fig 8C). As with the rough MABC, no significant differences were observed between NTM alone and THP-1 cells that were exposed to NTM. However, significant differences were observed between 4-OI treated NTM and 4-OI treated THP-1 cells exposed to NTM, indicating that the observed differences in NTM levels were a result of both the 4-OI and THP-1 cells and that 4-OI enhances THP-1 cells’ ability to clear smooth MABC (Fig 8C).

**Fig 9:**
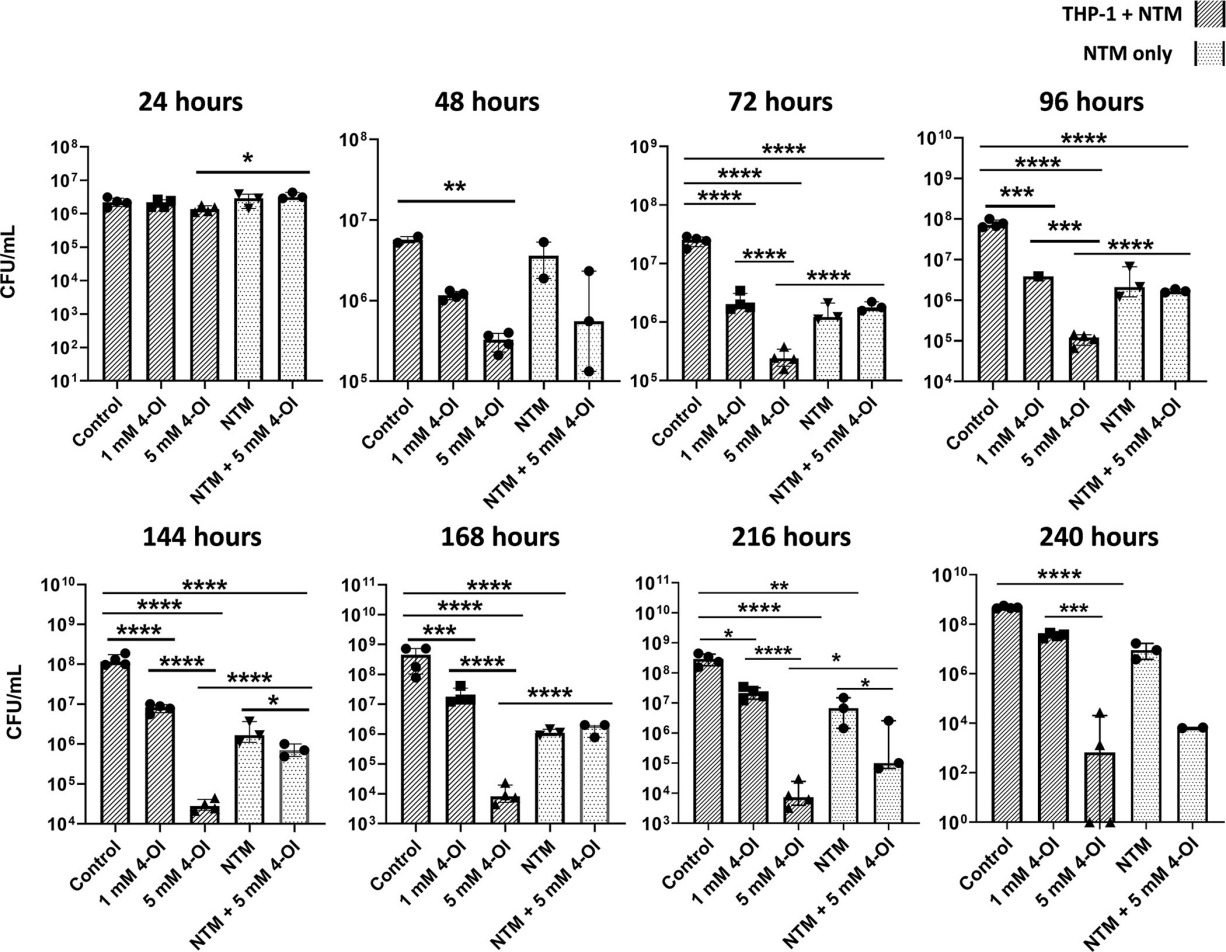
4-OI enhances the clearance of MAC by THP-1 cells. Clinically isolated *M*. *avium* 813 was added to differentiated THP-1 cells at an MOI of 15:1. 4-OI was added at the indicated concentrations and THP-1 cells with NTM were incubated at 37°C at 5% CO_2_ for the indicated lengths of time while the samples with NTM alone were incubated at 37°C. Control refers to THP-1 cells exposed to NTM without the addition of any 4-OI. Statistical tests were performed using Two-Way ANOVA; Sidak’s multiple comparisons test, on log transformed data. Error bars indicate median with interquartile range and asterisks indicates a significant difference between the compared samples. **** p ≤ 0.0001. n = 1–4.

For THP-1 cells exposed to *M*. *avium* 813 at a MOI of 10:1 and treated with 4-OI, significant differences in the number of surviving MAC were observed after 48 hours, while a dose response (i.e. a significant difference between the surviving NTM in THP-1 cells treated with 1 mM or 5 mM 4-OI) was not observed until the 72 hour time point and were consistent through the 96 hour time point. At the 144, 168, and 216 hour time points, 4-OI treated dose effects were still present, however, significant differences were observed between the number of surviving NTM in the control THP-1 cells, and the NTM alone in RPMI media (Fig 9). At the final 240 hour time point, significant differences were only observed when comparing the THP-1 control cells exposed to MAC to the 5 mM 4-OI treated THP-1 cells exposed to MAC (Fig 9).

## Discussion

We have demonstrated that itaconate/IA can serve as a potent growth inhibitor of clinical strains of *M*. *avium* and *M*. *abscessus* when cultured in minimal media supplemented with SCFAs, a primary carbon source for NTM bacteria when replicating and persisting inside of a host phagosome [55,58,69]. We also observed that IA (10 mM) was able to prevent the growth of NTM in complete 7H9 media (supplemented with OADC). Secondly, we have demonstrated that IA’s inhibition of NTM is pH dependent as 100 mM of pH = 7 IA was unable to inhibit the growth of *M*. *abscessus* 253 and *M*. *avium* 813. Third, our study also showed that a more acidic pH enhances the inhibitory effects of IA, as smaller concentrations of IA were needed to inhibit the growth of *M*. *abscessus* 253 and *M*. *avium* 813 when these NTM strains were cultured in more acidic (pH = 6.1) 7H9 minimal media. We also were able to show that IA can inhibit the activity of *M*. *abscessus* ICL in vitro, and lastly, that adding itaconate (4-OI) to THP-1 cells enhances clearance of phagocytosed NTM. The observation that there was significantly less MAC in the RPMI containing bacteria alone vs the THP-1 cells exposed to MAC is surprising but is likely due to a higher level of evaporation of the RPMI samples alone as they were stored in a different incubator from the THP-1 samples for the duration of the experiment.

Less IA was required to inhibit the growth of most NTM isolates supplemented with butyrate, a four carbon SCFA. Since NTM must break down butyrate via β-oxidation before it can be utilized [70–72], select enzymes in this process may be inhibited by the more acidic pH, specifically enzymes needed to catabolize longer SCFAs. While much of the β-oxidation machinery in mycobacteria are redundant, encoding multiple enzymes for each step in β-oxidation, in the case of MTB, some such as electron-transferring-flavoprotein dehydrogenase (EtfD) are essential to SCFA utilization [71]. If these enzymes are disrupted by a more acidic pH, that could account for the lack of proliferation. Another possibility is that the more acidic pH of the minimal media prevents transport of butyrate into the bacteria. In MTB, the protein lipid uptake coordinator A (LucA) facilitates fatty acid and cholesterol uptake into the bacteria by stabilizing protein subunits of the Mce1 and Mce4 transporters [73]; a similar system is likely present in NTM. It is also possible that the more acidic pH in the minimal media results in a conformational change to the albumin included in the media to prevent SCFA toxicity by slowing their release [74–76]. Studies examining mycobacteria using SCFAs as a food source have documented that SCFAs can be harmful to the bacteria if utilized too quickly due to the buildup of toxic intermediates and this may have introduced an increased level of complexity in our study [69]. A conformational change in albumin could therefore result in butyrate release at a rate that is harmful to the NTM. Interestingly, butyrate also has the ability to enhance macrophage activity against pathogens, including *M*. *bovis* [77–79]. Butyrate may also have a synergistic effect in our study as we observed a low dose of IA was effective when used in conjunction with butyrate (Fig 6).

However, two MAC clinical isolates proliferated in 7H9 MM supplemented with butyrate and 5 mM IA. The reasons for this are unclear but are likely strain specific as MAC isolates are fairly genetically diverse [80,81]. There may be some genetic features possessed by these two MAC isolates (such as variation in their β-oxidation machinery), that allow these NTM to still proliferate even in an acidic pH. The importance of these differences between MAC isolates remains to be elucidated but will be a point of focus in future studies. It is also worth noting that none of the tested NTM isolates were capable of itaconate dissimilation to utilize it for growth. We did not detect Rv2498c β-hydroxyacyl-CoA lyase, the enzyme responsible for itaconate dissimilation in MTB, in our sequenced strains from this study.

While the amount of IA needed to inhibit growth (5–10 mM) in our study exceeds what is commonly detected in human samples (60 uM) [51,61], other studies have demonstrated that immune cells may have the ability to sequester itaconate, increasing the concentration of itaconate in certain cellular compartments [45]. Additionally, since our data has shown that the effectiveness of IA is enhanced in more acidic conditions, the lower overall concentrations of itaconate detected from airway samples and macrophages (compared to that of a mouse where the levels of itaconate are higher) could still serve an antimicrobial role against NTM [45,51]. While the mechanism of IA’s increased effectiveness in a more acidic pH is unknown, it is likely due in part to an increased ability of itaconate/IA to enter NTM cells [47] and will be a point of focus in future studies. Moreover, other studies have found that the antimicrobial effect of itaconate/IA is dependent upon the pH of the media and that itaconate/IA’s effects are enhanced in a more acidic pH media, particularly under nutrient poor conditions for both *E*. *coli* and *Salmonella enterica* serovar Typhimurium [61]. Coupled with IA’s lost ability to inhibit NTM at a neutral pH, this would suggest that the pH of the medium IA is in has large effects on the compounds ionization state. This observation has major implications for mycobacteria in particular as the bacteria are often able to prevent the full maturation and acidification of the phagosome via use of the type vii secretion system, allowing them to escape the phagosome and enter the cytosol of the host cell [82,83]. If a sufficient amount of itaconate is located in the airway and/or readily available to the macrophages encountering mycobacteria it could potentially serve to completely halt and/or eliminate any bacterial proliferation. A recent study by Kim et al. found that dimethyl itaconate (DMI) could be a promising candidate for host-directed therapeutics against both MTB and NTM due to its ability to activate multiple innate immune responses [84].

## Conclusions

As previously stated, the pH level of the airway is generally considered to be more acidic in pwCF [62–64]. This increased acidity could potentially be enhancing the inhibitory effects of itaconate/IA, helping to prevent NTM infections and/or persistent colonization from occurring. Moreover, we have observed that itaconate levels are decreased prior to and during NTM infection in pwCF [60]. These findings suggest that itaconate could be of salient in host defense against NTM infection in pwCF. While deficiencies in immune cell function such as macrophages are well documented in pwCF, at this time it is not known if CF macrophages are deficient in itaconate production or if increased itaconate levels would lead to better control of NTM infections [85]. Additionally, while metabolomics analyses of the CF airway have been able to determine the overall amount of itaconate and other metabolites in the airway, an open question remains as to the overall distribution of those metabolites in the CF airway. If the distribution of metabolites such as itaconate is rather uneven, it could create small niche areas within the sputum and the airway that consequently become immune privileged sites where NTM and/or other pathogenic bacteria can proliferate with relative impunity. Future studies will aim to expand upon this work by examining the mechanism of interaction between NTM and macrophages form both pwCF and otherwise healthy individuals, and also aim to examine the relationship between itaconate and anaerobic bacteria that are frequently found in the airway.

## Supporting information

S1 Fig MAC and MABC are able to proliferate in acidic (pH = 4.8) 7H9 minimal media. (TIF)

S1 File Raw data used for generating figures. (XLSX)
