# Supplementary figures and images for: Itaconic acid inhibits nontuberculous mycobacterial growth in pH dependent manner while 4-octyl-itaconic acid enhances THP-1 clearance of nontuberculous mycobacteria in vitro
Source: PLoS One. 2024 May 10;19(5):e0303516. doi: 10.1371/journal.pone.0303516 (PMC11086914; doi:10.1371/journal.pone.0303516)

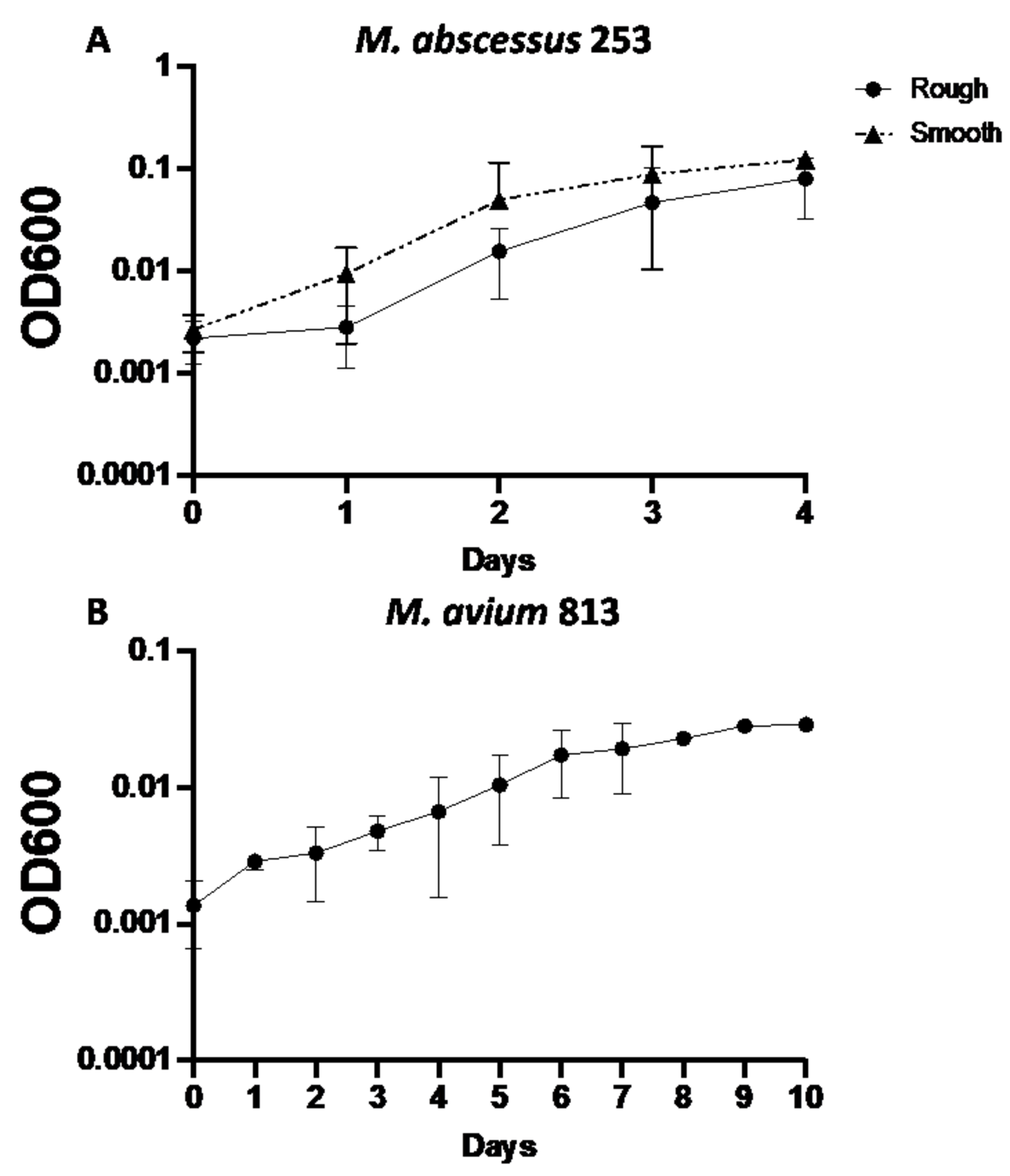

Supplement: S1 Fig — (TIF) [file pone.0303516.s001.tif]
